# Analyzing the super-resolution characteristics of focused-spot illumination approaches

**DOI:** 10.1117/1.JBO.25.5.056501

**Published:** 2020-05-21

**Authors:** Jiun-Yann Yu, Venkatalakshmi Narumanchi, Simeng Chen, Jian Xing, Stephen R. Becker, Carol J. Cogswell

**Affiliations:** a University of Colorado Boulder, Department of Electrical, Computer and Energy Engineering, Boulder, Colorado, United States; b University of Colorado Boulder, Department of Applied Mathematics, Boulder, Colorado, United States

**Keywords:** biomedical imaging, fluorescence microscopy, super-resolution microscopy, image deconvolution

## Abstract

**Significance:** It is commonly assumed that using the objective lens to create a tightly focused light spot for illumination provides a twofold resolution improvement over the Rayleigh resolution limit and that resolution improvement is independent of object properties. Nevertheless, such an assumption has not been carefully examined. We examine this assumption by analyzing the performance of two super-resolution methods, known as image scanning microscopy (ISM) and illumination-enhanced sparsity (IES).

**Aim:** We aim to identify the fundamental differences between the two methods, and to provide examples that help researchers determine which method to utilize for different imaging conditions.

**Approach:** We input the same image datasets into the two methods and analyze their restorations. In numerical simulations, we design objects of distinct brightness and sparsity levels for imaging. We use biological imaging experiments to verify the simulation results.

**Results:** The resolution of IES often exceeds twice the Rayleigh resolution limit when imaging sparse objects. A decrease in object sparsity negatively affects the resolution improvement in both methods.

**Conclusions:** The IES method is superior for imaging sparse objects with its main features being bright and small against a dark, large background. For objects that are largely bright with small dark features, the ISM method is favorable.

## 1 Introduction

Using a tightly focused light spot for illumination has been one of the most significant advancements in the history of optical microscopy. Since the mid-20th century, this technique has brought about the invention^1^ and the reinvention^2^ of confocal microscopy, while also introducing new microscopes that instantly acquire digital images with adjustable pixel sizes and pixel numbers.^3^ Based on these new microscope designs, many novel scientific tools have emerged and enabled ground-breaking discoveries, including fluorescence correlation spectroscopy,^4^^,^^5^ optical coherence tomography,^6^ and super-resolution microscopy.^7^^,^^8^ In the past few years, much attention has been drawn to a particular reinvented confocal imaging technique^9^ called image scanning microscopy (ISM),^10^ due to its extraordinary photon efficiency as well as its confocal super-resolution effect.^11^

**Fig. 1:**
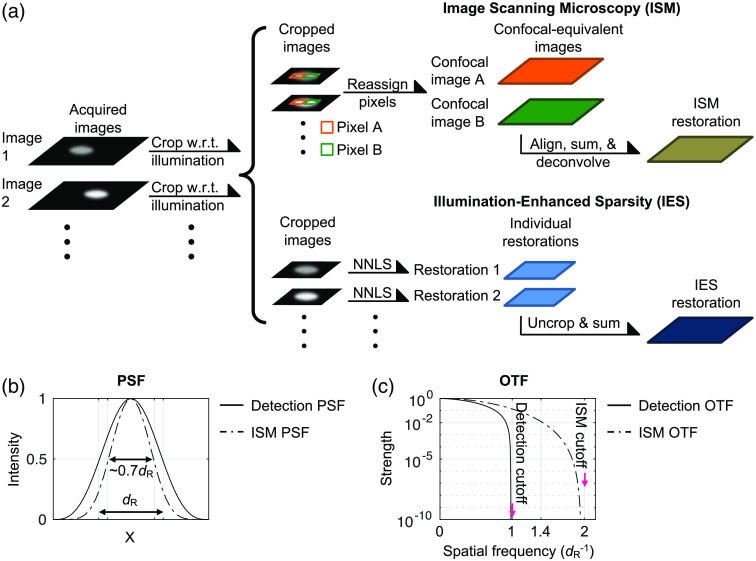
(a) Workflows of the ISM and IES methods. (b) PSF and (c) OTF plots for the ISM images (dashed lines) and the detection part of the optical system (solid lines). $d_{R}$ is the Rayleigh resolution distance, estimated as 0.6λ/NA, where λ is the light wavelength and NA is the optical system numerical aperture.

The original ISM method uses the objective lens to create a tightly focused illumination spot, steps this spot across the object, and acquires one descanned image at each step.^10^ For images acquired without a descan arrangement, each acquired image is cropped with respect to its illumination spot position.^12^ For these descanned or cropped images, each pixel is considered equivalent to a pinhole detection in a confocal microscope and is reassigned to create a particular confocal image [Fig. 1(a)]. Consequently, many confocal images are generated from a complete lateral scan.

In particular, it has been shown that these confocal images are laterally displaced versions of one another.^9^^,^^11^ With appropriate alignment, these confocal images can be summed to increase the signal-to-noise ratio (SNR) while maintaining the superior lateral resolution of a small-pinhole confocal detection. In terms of lateral resolution, the effective point spread function (PSF) of an ISM image can be derived by spatially multiplying the illumination PSF and the detection PSF of the optical system, which makes the effective PSF of ISM sharper than the detection PSF by a factor of $∼\sqrt{2}$ or ∼1.4 [Fig. 1(b)].^9^ We can also observe such a resolution improvement in the optical transfer function (OTF), i.e., the Fourier transform of the intensity PSF.^13^ Although the PSF multiplication described above makes the ISM OTF cutoff frequency nearly twice as large as the detection OTF cutoff frequency,^9^^,^^10^ the strength for spatial frequency higher than $1.4d_{R}^{-1}$ is very weak [Fig. 1(c)] and therefore can be lost with the presence of various noise sources. In other words, the ISM image obtained from the pixel reassignment procedure visually exhibits a ∼1.4-fold resolution enhancement over the Rayleigh resolution limit,^9^ and to fully retrieve the information within the doubled OTF cutoff frequency requires additional deconvolution approaches, such as Wiener deconvolution^10^ or non-negative least-squares (NNLS) deconvolution.^12^^,^^14^

Interestingly, although the twofold resolution improvement is often regarded as one of the most important features of ISM,^10^^,^^15^ it has been demonstrated in much earlier studies that more than twice the Rayleigh resolution limit can be achieved by applying certain nonlinear deconvolution algorithms directly to the conventional widefield images. For instance, Frieden et al. experimentally demonstrate that a two-line object with a gap of one third of the Rayleigh resolution limit can be correctly resolved using a positively constrained deconvolution algorithm.^16^ Donoho et al.^17^ argued that the object sparsity (referred to as near-blackness in their work) is critical to such resolution enhancement and derived that the resolution enhancement increases with the object sparsity. Donoho et al. quantified object sparsity as, for a discrete numerical object, the ratio of the number of its near-zero elements to the number of its total elements. We note that such a definition considers only how many “bright” elements there are, ignoring their geometrical arrangement. More importantly, as shown in these earlier studies, when the object sparsity is critical to resolution enhancement, the deconvolution algorithms in use do not implement a mathematical constraint to favor sparse solutions.^16^^,^^17^

Based on such sparsity-associated resolution enhancement, the authors recently proposed utilizing focused-spot illumination to physically enhance object sparsity and demonstrated substantial resolution enhancement through image deconvolution without implementing any sparsity constraint.^18^ This illumination-enhanced sparsity (IES) method enhances object sparsity by creating a tightly focused illumination spot through the objective lens and images a small spot at each position during scanning, as is done in ISM [Fig. 1(a)]. Therefore, for each acquired image, the object is nearly black outside of the illumination spot. The acquired images are cropped with respect to the illumination spot positions and individually deconvolved with an NNLS algorithm to create individual restorations. In the end, the individual restorations are shifted to their corresponding positions in the object space and integrated to create a complete restoration of the object [Fig. 1(a)]. The NNLS algorithm used for deconvolution assumes only a physical property that the fluorescent photons emitted from the object are spatially incoherent, meaning that the signals from different emitters add linearly when detected by a camera, and all elements in the object are non-negative. As mentioned above, the deconvolution algorithm does not implement a sparsity constraint and assumes no knowledge about the illumination. It is experimentally demonstrated that the IES method can achieve more than twofold resolution improvement over the Rayleigh resolution limit.^18^

Considering the observations and derivations in previous reports,^16^^,^^17^ it is natural to predict that the signal level and object sparsity will affect the resolution enhancement in IES because of its usage of non-negatively constrained deconvolution algorithms. Intriguingly, as mentioned earlier, many ISM realizations also utilize non-negatively constrained deconvolution algorithms such as NNLS deconvolution^12^^,^^14^ to achieve beyond the ∼1.4-fold resolution enhancement gained from pixel reassignment. Therefore, it is also reasonable to expect a decrease in ISM resolution when the image SNR or object sparsity drops.

However, it is often assumed or stated in research articles^12^^,^^14^^,^^19^^,^^20^ as well as in commercial product descriptions^15^ that ISM can always achieve the maximal twofold resolution enhancement without accounting for any other imaging factors. As such, we find it worthwhile to fully investigate the accuracy of this conventional wisdom. In addition, how these two super-resolution methods respond to factors such as the image SNR and object sparsity, which can vary greatly in different biomedical imaging scenarios, has not yet been carefully examined.

The goals of this paper are therefore to evaluate and compare the resolution enhancement and restoration quality of the ISM and IES methods as we manipulate the image SNR and object sparsity. For fair comparisons, we generate the same image datasets from a series of computational simulations and a biological imaging experiment and feed them to the ISM and IES methods. In the following sections, we describe how the image datasets are generated and compare the restorations of the two methods.

## 2 Formulation of Numerical Simulation

It is technically challenging to use experimentally collected image data for comparing the two super-resolution methods with controlled object factors. This is because manufacturing precise fluorescent objects that are sufficiently stable to endure repetitive imaging under various conditions without significant signal decay can be very complicated and expensive. Consequently, we find it more economic and accurate to numerically generate synthetic image data for comparisons.

### 2.1 Generating Synthetic Image Data

We start formulating the synthetic data generation by considering noiseless image formation in a 1:1 conventional widefield imaging system, where the noiseless image $I^{\text{noiseless }\mathrm{img}}(s)$ can be derived as

$$I^{\text{noiseless }\mathrm{img}}(s)=I^{\mathrm{PSF}}(s)⊗[I^{\mathrm{illm}}(s)\cdot I^{\mathrm{obj}}(s)].$$ (1)

Here s is a two-dimensional vector $\left(s_{x},s_{y}\right)$ representing the lateral coordinates on the image or object plane, $I^{\mathrm{PSF}}$ is the detection PSF of the optical system, ⊗ is the convolution operation, $I^{\mathrm{illm}}$ is the illumination function, · is the inner product operation, and $I^{\mathrm{obj}}$ is the object.

The image acquisition in ISM and IES consists of stepping the illumination spot across the object; therefore, the k’th acquired image $I^{k-\mathrm{img}}(s)$ is, considering photon shot noise,

$$I^{k\text{-}\mathrm{img}}(s)=\text{Poiss}\{I^{\mathrm{PSF}}(s)⊗[I^{k\text{-}\mathrm{illm}}(s)\cdot I^{\mathrm{obj}}(s)]\}.$$ (2)

Here $I^{k\text{-}\mathrm{illm}}$ is the k’th illumination function of the optical system; Poiss(·) generates a Poisson random number for each input element, with the mean equal to the element value. We do not include the camera readout noise in Eq. (2) because the readout noise in high-sensitivity cameras used for advanced fluorescence microscopy is typically much smaller than the photon shot noise. For numerical computation, we formulate Eq. (2) as

I^k-img=Poiss[H^PSF×(I^k-illm·I^obj)], (3)

where column vectors I^k-img, I^k-illm, and ${\hat{I}}^{\mathrm{obj}}$ are discrete and vectorized $I^{k\text{-}\mathrm{img}}(s)$, $I^{k\text{-}\mathrm{illm}}(s)$, and $I^{\mathrm{obj}}(s)$, respectively. Here × is the matrix product operation and H^PSF is a matrix with its j’th column being a vectorized detection PSF centered at position $s_{j}$. For the simulations presented in this work, we set both the camera pixel size and the scanning step size to be a fourth of the Rayleigh resolution distance or $d_{R}$, which is estimated as 0.6λ/NA, where λ is the fluorescence emission wavelength and NA is the detection numerical aperture of the optical system.

After the acquired images are generated, we crop each with respect to its illumination position, such that the illumination is effectively placed in the center of the cropped image $I^{k\text{-}\mathrm{img}\text{ crop}}$. This can be formulated as

$$I^{k\text{-}\mathrm{img}\;\mathrm{crop}}(s^{\prime })=I^{k\text{-}\mathrm{img}}(s^{\prime }+v_{k}),\;\text{for }|s_{x}^{\prime }|\leq \frac{\ell }{2},\;|s_{y}^{\prime }|\leq \frac{\ell }{2}.$$ (4)

Here $v_{k}$ is the central position of the k’th illumination function and ℓ is the size of the cropped images, set as $8d_{R}$ in our simulations. We then use the ISM and IES methods individually to process these cropped images.

### 2.2 Image Processing

#### 2.2.1 ISM method

In ISM, the pixels in the cropped images are considered equivalent to individual confocal detectors. Therefore, we reassign the pixel at $s_{\alpha }^{\prime }$ in $I^{k\text{-}\mathrm{img}\text{ crop}}$ to $v_{k}$ in a new confocal image $I^{\alpha \text{-}\mathrm{CFimg}}$, such that

$$I^{\alpha \text{-}\mathrm{CFimg}}(v_{k})=I^{k\text{-}\mathrm{img}\text{ crop}}(s_{\alpha }^{\prime }).$$ (5)

In particular, we only reassign pixels within an Airy unit in the cropped images, meaning that we require $|s_{\alpha }^{\prime }|\leq d_{R}$ for the pixels to be reassigned. The effective PSFs for pixels outside of this range become irregular and are no longer ideal for confocal imaging. For the α’th reassigned confocal image $I^{\alpha \text{-}\mathrm{CFimg}}$, it can be shown that it has a spatial shift of $∼-s_{\alpha }^{\prime }/2$ relative to the confocal image formed by the central pixel in the cropped images.^9^^,^^11^ This allows us to align these confocal images with appropriate counter shifts and integrate them to create a high-SNR ISM image $I^{\mathrm{ISMimg}}$ as

$$I^{\mathrm{ISMimg}}(v_{k})=\underset{\alpha }{\sum }I^{\alpha \text{-}\mathrm{CFimg}}(v_{k}-\frac{s_{\alpha }^{\prime }}{2}).$$ (6)

The numerical computation for Eq. (6) requires interpolation because $\left(v_{k}-\frac{s_{\alpha }^{\prime }}{2}\right)$ sometimes locates in the middle of actual data points. We use MATLAB’s interp2 and its “cubic” method for such interpolation.

As mentioned, many ISM realizations further utilize deconvolution methods to fully exploit the expanded OTF; these methods include Wiener deconvolution,^10^^,^^21^ Richardson–Lucy deconvolution,^22^ and NNLS deconvolution.^12^^,^^14^^,^^23^^,^^24^ For our ISM restorations, we perform all three deconvolution methods on $I^{\mathrm{ISMimg}}$ and find that each of the methods has its own strengths. Figure S1 in the Supplementary Material shows a comparison among the three methods. In general, we find that the results from NNLS deconvolution are consistently in good quality across all of the different test objects and that its software does not require judicious parameter settings by experienced microscopists (Sec. 1 and Fig. S2 in the Supplementary Material). Consequently, in the main figures in this paper, we only present results from the NNLS deconvolution. The software we use to perform NNLS deconvolution on $I^{\mathrm{ISMimg}}$ is an ImageJ plugin for iterative deconvolution,^23^ which is used in the ISM realizations developed by York et al.^12^ and Azuma and Kei.^14^ In all presented simulations, we use the “WPL” method, which is an NNLS solver preconditioned by a Wiener filter,^24^ along with the same other settings and the default stopping conditions as in the previous works.

For all three deconvolution methods, the PSF is a required input, and we note that the effective PSF for $I^{\mathrm{ISMimg}}$ is no longer the detection PSF of the optical system. As mentioned earlier, we can obtain the effective PSFs for $I^{\mathrm{ISMimg}}$ by multiplying the illumination and detection PSFs.^9^^,^^11^ Alternatively, we can obtain the effective ISM PSF by performing the calculation from Eq. (3) through Eq. (6) assuming the object $I^{\mathrm{obj}}$ is a single dot (i.e., all but one of the elements in I^obj are zero), with the Poisson operation in Eq. (3) removed. We implemented both methods and verified that they give identical PSFs.

### 2.3 IES Method

In IES, we feed each of the cropped images to NNLS deconvolution individually [Fig. 1(a)]. The NNLS deconvolution finds a minimum for the following problem:

minI^k-IES‖H^PSF×I^k−IES−I^k−img crop‖2,subject to  I^k-IES≥0 (7)

and returns I^k-IES as the vectorized restoration for the k’th cropped image. The inequality is taken component-wise, and ‖·‖ is the Euclidean norm (sum-of-squares). We note that the PSF matrix H^PSF here is derived directly from the detection PSF of the optical system. In all of the simulations presented in this paper, we use MATLAB’s quadprog with its “interior-point-convex” method and default stopping conditions for the NNLS deconvolution.

After all of the individual restorations are generated, we uncrop each restoration by positioning the individual restoration to its original crop location in $I^{k\text{-}\mathrm{img}}$ and then integrate all of the uncropped individual restorations to obtain the complete IES restoration, or $I^{\mathrm{IES}}$, such that

IIES(s)=∑k,|vkx−sx|≤ℓ2&|vky−sy|≤ℓ2Ik-IES(s−vk). (8)

Here $I^{k\text{-}\mathrm{IES}}$ is the individual NNLS restoration for the k’th cropped image.

In terms of the computation time for image deconvolution, the ISM method typically requires less than a second on a personal computer for images acquired from 100×100 scanning steps. This can be further accelerated for real-time display on a dedicated computer. For the same amount of image data, the computation time of the IES method can range from several seconds to a few hours, depending on the NNLS algorithm and the hardware architecture in use.

## 3 Results

**Fig. 2:**
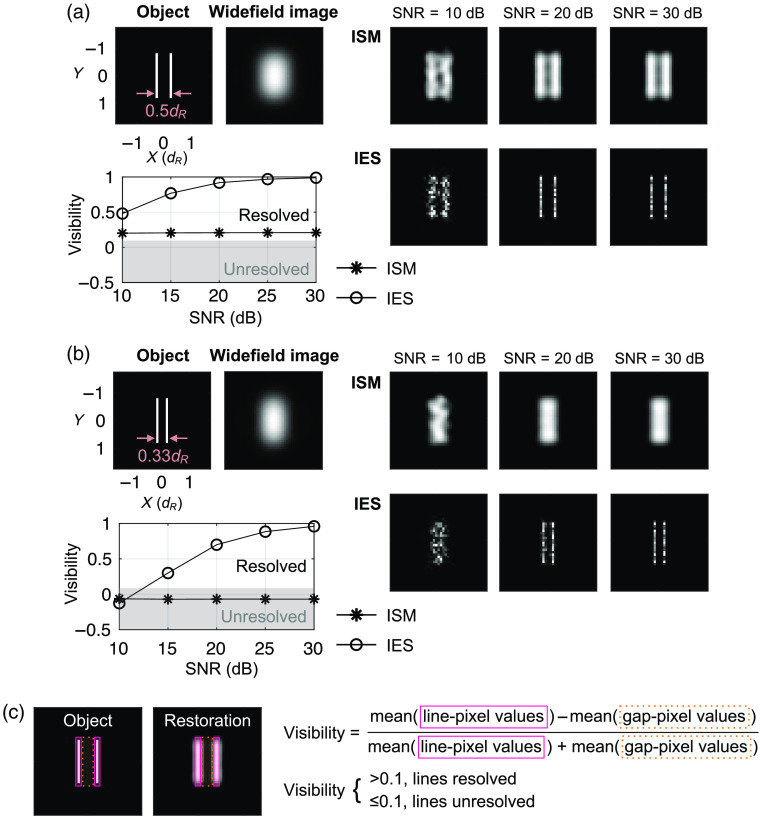
ISM and IES restorations at various SNRs. (a) Representative restorations for two lines at a $0.5d_{R}$ separation and a visibility-SNR plot. Each data point in the plot is the mean of nine independent trials. (b) The representative restorations and visibility-SNR plot for a $0.33d_{R}$ separation. (c) Definition of restoration visibility: the mean value of the pixels overlapping with the bright lines in the object divided by the mean value of the pixels overlapping with the gap(s) in between bright lines in the object. We set 0.1 visibility as the resolution criterion, which is the approximate visibility when two lines are separated by $d_{R}$ in conventional widefield imaging.

**Fig. 3:**
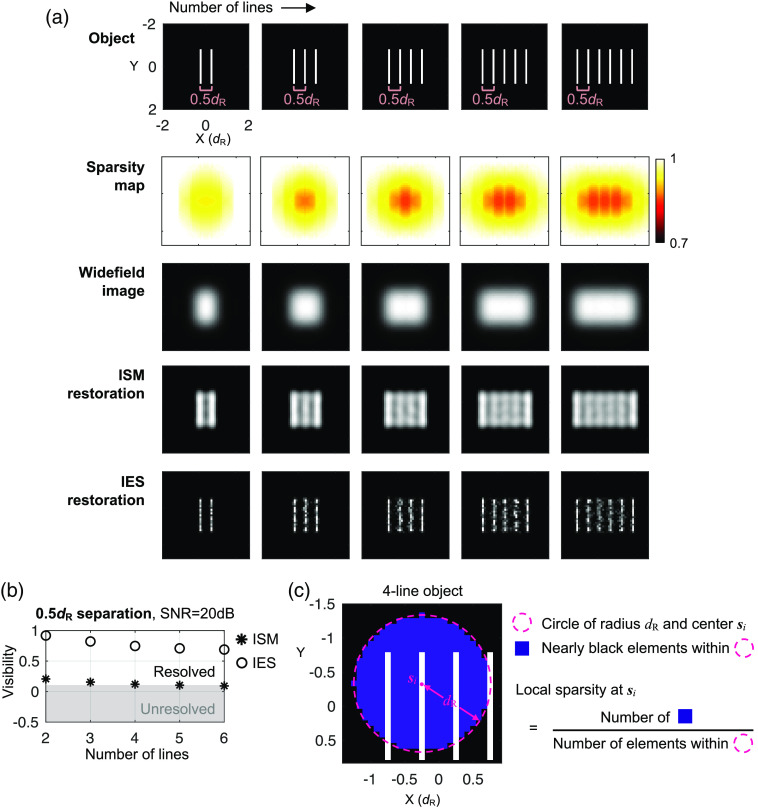
(a) Representative ISM and IES restorations and (b) restoration visibility for objects of various numbers of lines at $0.5d_{R}$ separations. All simulations assume a 20-dB image SNR. Each visibility data point is the mean of nine independent trials. (c) Illustration of the local sparsity definition.

**Fig. 4:**
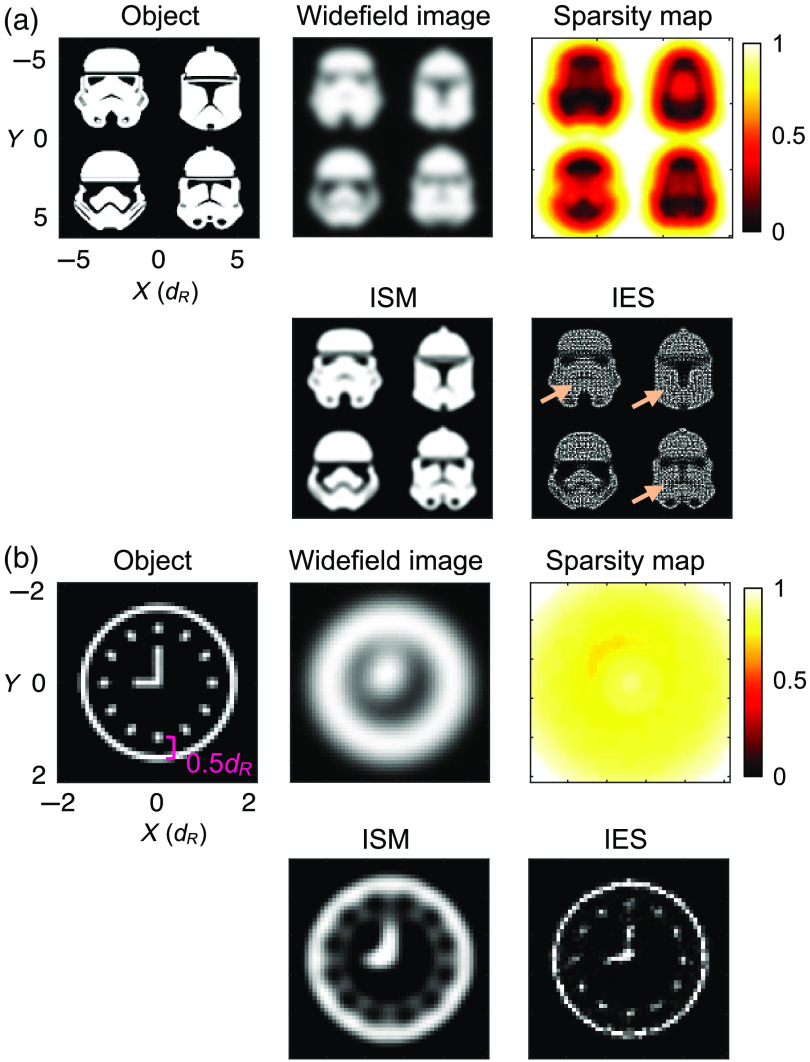
ISM and IES restorations of arbitrary objects consisting of (a) nonsparse patterns and (b) a sparse pattern. Arrows in the IES restoration in (a) indicate the dark features that are poorly resolved.

**Fig. 5:**
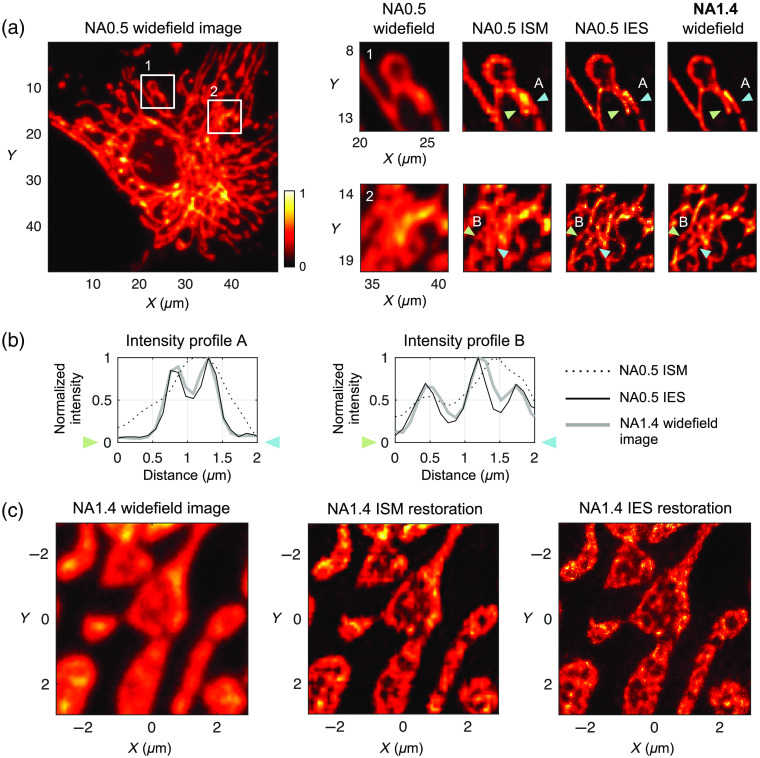
Biological fluorescence imaging of BPAE cells. The object consists of fluorescently labeled mitochondria, with ∼600  nm emission wavelengths. (a) NA0.5 conventional widefield image, zoom-in restorations in white boxes 1 and 2, and NA1.4 conventional widefield images. $d_{R}$ for the NA0.5 imaging is ∼0.8  μm. (b) Intensity line plots for linear regions indicated by the pairs of arrows in (a). (c) Another region of the same sample imaged with an NA1.4 objective and the corresponding ISM and IES restorations.

Using the synthetic image data generated by the above simulations, we compare the restorations of the two methods as we vary the image SNR (Fig. 2) and object sparsity (Fig. 3). We further examine the robustness of the two methods by imaging objects of arbitrary shapes (Fig. 4) and a biological sample (Fig. 5).

### 3.1 Resolution Enhancement versus Image SNR

To the best of our knowledge, there has been no previous report on whether or not the twofold resolution enhancement of ISM depends on the SNR of the acquired images. For the IES method, by contrast, Donoho et al. argued that, as long as the image SNR is sufficient, objects of high sparsity can always be resolved by NNLS deconvolution, although the required SNR may be prohibitively high in practice.^17^ Such an argument predicts that certain objects unresolved at low SNRs may become resolved at high SNRs.

Figures 2(a) and 2(b) show two series of ISM and IES restorations with the image SNR equal to 10, 20, and 30 dB. The two lines in the object are separated by $0.5d_{R}$ in Fig. 2(a) and $0.33d_{R}$ in Fig. 2(b). Because the SNR for a given Poisson random variable is the square root of its mean, we define the image SNR of a synthetic image dataset as

SNR=10 log10max[(M1,M2,…,Mn)]  dB,where  Mk=max(I^k−noiseless img). (9)

Here max[·] is a function that returns the maximum element value in a vector, n is the total number of scanning steps, and I^k-noiseless img is the vectorized noiseless image for the k’th scanning step. For each of the separation distances, we also show a plot of restoration visibility versus the image SNR. We define the restoration visibility in a similar manner as the fringe visibility in interferometry to measure how well the objects are restored [Fig. 2(c)].

In Figs. 2(a) and 2(b), we indeed observe that the ISM resolution reaches a twofold resolution enhancement over the Rayleigh resolution limit and not beyond. The IES method, by contrast, is able to resolve the two-line object of a $0.33d_{R}$ separation, which requires at least threefold resolution enhancement over the Rayleigh resolution limit to resolve. Our further simulations show that the IES method can resolve a $0.17d_{R}$ separation with a ∼0.5 visibility at a 30-dB SNR (data not shown).

In the visibility-SNR plots in Figs. 2(a) and 2(b), the ISM restoration visibility shows little change as the SNR increases. For example, for the two-line object of a $0.5d_{R}$ separation, the ISM restoration visibility improves just 3% as we increase the SNR from 10 to 30 dB. In contrast, the IES restoration visibility improves significantly as the SNR increases. Similar trends are also observed when we further shorten the separation distance to $0.25d_{R}$ and $0.17d_{R}$ (data not shown). Such an observation agrees with the prediction in the report of Donoho et al. that when the object is sparse enough, it can be resolved by the NNLS deconvolution as long as the image SNR is sufficiently high.^17^

For most fluorescent samples, the fluorophore labeling density fluctuates at a certain degree; therefore, the fluorescence intensity on the object structures is not a constant. To examine whether the restoration visibility of two methods is affected with the presence of such intensity variation, we further simulate the restorations where the object line intensity varies at different degrees (Sec. 2 and Fig. S3 in the Supplementary Material).

### 3.2 Resolution Enhancement versus Object Sparsity

We next turn to the effect of object sparsity. To examine how the object sparsity affects the restorations of the two methods, we manipulate the object sparsity by designating different numbers of lines in the objects with a fixed separation distance between them.

Figure 3(a) shows ISM and IES restorations from objects that have two to six lines, with a $0.5d_{R}$ separation distance and a 20-dB image SNR. To visualize the spatial variation of object sparsity, we plot a local sparsity map for each object. Here we define the local sparsity I^sparsity at position $s_{i}$ in the object as the fraction of nearly black elements within a range of a radius $d_{R}$ surrounding the position [Fig. 3(c)]. This can be written as

I^isparsity=∑j,|si−sj|≤dRBj∑j,|si−sj|≤dR1,Bj={1,if  I^jobj<0.01  max(I^obj)0,otherwise. (10)

Figure 3(a) shows that the the ISM restoration quality gradually drops as the number of lines increases, inconsistent with the common assumption that the twofold resolution enhancement of ISM is independent of object sparsity. For the six-line object, although ISM vaguely resolves the gaps between the lines, it is difficult to determine whether the restoration shows a solid bright rectangle or separated lines without knowing the object composition in advance. To ensure that the visual trend we observe here is systematic, we repeat the simulation 9 times for each object and plot the mean visibility of these nine independent trials versus the number of object lines in Fig. 3(b). The visibility plot suggests that the NNLS deconvolution in the ISM method is indeed sensitive to the decrease in object sparsity. This trend is also observed when we use Richardson–Lucy deconvolution for ISM [Fig. S1(b) in the Supplementary Material].

For the IES restorations, as we predict, we see a similar trend of declining restoration visibility as in the ISM restorations. In further simulations at a 20-dB image SNR, we find that the IES method can resolve only up to three lines of a $0.33d_{R}$ separation and only up to two lines of a $0.25d_{R}$ separation (data not shown). In addition, we find that similar trends of declining visibility can be observed in other geometrical structures (Fig. S4 in the Supplementary Material). We also examine the effect of sparsity decrease caused by the presence of a fluorescent background, which occurs frequently in biological fluorescence microscopy, and show that it deteriorates the restoration visibility in both ISM and IES methods (Fig. S5 in the Supplementary Material).

### 3.3 Robustness of Resolution Enhancement for Arbitrary Objects

We further examine the super-resolution capability of the ISM and IES methods in a more realistic setting where the objects are in arbitrary shapes. Figure 4(a) shows the restorations of an object consisting of four nonsparse patterns with their distinctive features defined by a small number of nearly black pixels in regions where the local sparsity is close to zero. In this case, the ISM restoration faithfully preserves many of the dark features in the object, whereas the IES restoration shows strong intensity fluctuations in areas where the object is uniformly bright. Such an artifact in the IES restoration makes the dark features in the object patterns almost invisible.

Figure 4(b) shows the restorations of a clock-pattern object with its features being, in contrast, decided by a few bright pixels. Although the distance between the hour markings and the clock frame is $∼0.5d_{R}$, we find that the ISM restoration fails to resolve these gaps clearly. In addition, the ISM restoration falsely creates a dim but visible inner circle connecting all of the hour markings, which in turn makes the restoration visually resemble a rotary dial (of antique telephones) instead of a clock. In the IES restoration in Fig. 4(b), we again observe intensity fluctuations, which is likely responsible for the broken minute hand in the restoration. Aside from this, the IES restoration faithfully preserves the hour markings and the clock frame; therefore, we can easily recognize the clock pattern in the restoration.

### 3.4 Fluorescence Imaging of a Biological Sample

Finally, we image a fluorescent biological sample to examine whether our simulation results are valid in real-world imaging experiments. In such experiments, it is technically challenging to obtain exact $I^{\mathrm{obj}}$, without which the restoration comparison can be highly subjective. To overcome this issue, we collect focused-spot illuminated images with a moderate NA (∼0.5) objective lens for ISM and IES restorations and use a much higher NA (∼1.4) objective lens to acquire a widefield image at the same location on the sample [Figs. 5(a) and 5(b)]. Here the NA1.4 widefield image serves as a super-resolving reference that has a resolution of more than twice the NA0.5 Rayleigh resolution limit. At a ∼600-nm emission wavelength, $d_{R}$ for NA0.5 imaging is ∼0.8  μm.

The biological sample in use is a commercially available fixed slide (FluoCell Prepared Slide #1, Thermo Fisher Scientific) for general observation purposes by fluorescence microscopy. The imaged fluorophore is MitoTracker Red CMXRos, labeling mitochondria in bovine pulmonary artery endothelial (BPAE) cells. We describe and illustrate the optical system used for image acquisition in Sec. 5 and Fig. S6 in the Supplementary Material.

In Fig. 5(a), the ISM restoration indeed shows substantial resolution improvement compared with the NA0.5 conventional widefield image, although certain details seen in the NA1.4 widefield image are not correctly resolved. The IES method, in contrast, shows a restoration almost identical to the NA1.4 widefield image. Figure 5(b) shows two intensity profile line plots taken across the regions indicated in the enlarged illustrations in Fig. 5(a) to confirm the quantitative accuracy of the IES restoration. The comparison of the two methods in Fig. 5(a) is consistent with the simulation results in Fig. 3(a), where the IES method produces more accurate restorations for objects of both high sparsity (e.g., two lines) and reduced sparsity (e.g., three to six lines).

Figure 5(c) shows the results of imaging this biological sample with an NA1.4 objective and focused-spot illumination. As observed in the case of NA 0.5, the ISM restoration shows substantial resolution improvement over the widefield image. The IES restoration further improves the resolution such that the internal compartments of the mitochondria are clearly resolved.

## 4 Discussions and Conclusions

Our work distinguishes two super-resolution methods that utilize the same image datasets acquired with focused-spot illumination. We demonstrate that their super-resolving restorations show significant differences in their maximum achievable resolution. In particular, we point out that the OTF expansion created by pixel reassignment in the ISM method is not necessarily an efficient approach to maximizing achievable resolution. It is worthwhile to note that this finding is in agreement with two previous reports on the deconvolution approaches for images acquired with focused-spot illumination.^19^^,^^20^ In both reports, all acquired images are deconvolved together using a generalized Richardson–Lucy deconvolution algorithm, without going through the pixel reassignment for OTF expansion, and it is shown that the results are at least equivalent to the ISM restorations.

In this report, for simplicity, we manipulate object sparsity by adding more structures or a uniform background into the object. In many biomedical applications, the object sparsity can also be affected by other factors, such as unspecific staining, photobleaching, and fluorophore blinking. In addition, object sparsity can be manipulated by stimulated emission or optical switching applied to switchable fluorescent probes. We believe that both the ISM and IES methods are subject to these factors and manipulations in terms of resolution enhancement, and we hope to investigate their individual and combined effects in future studies based on the methodology developed in this work.

Although we consider only the case of 2D imaging in this paper, we are currently investigating the super-resolution characteristics of the ISM and IES methods in three dimensions, as both methods can perform 3D imaging by utilizing 3D deconvolution. For the IES method, in particular, because the detection PSF of the optical system is nearly symmetrical along the depth dimension, at least two images, separated in depth, have to be taken at each illumination spot position. Such a requirement is analogous to using multifocal plane imaging for 3D particle tracking.^25^ An alternative strategy is to create an axially asymmetrical detection PSF via introducing a small amount of geometric aberration into the detection part of the optical system. This will then allow 3D deconvolution of a single image at each illumination spot position and is analogous to implementing PSF engineering for 3D localization microscopy.^26^^,^^27^ We hope to present our findings in 3D imaging in the near future.

To conclude, we find that the IES method is superior for imaging sparse objects, especially objects with main features being bright and small against a dark, large background. In such cases, IES restorations can often achieve resolution substantially higher than twice the Rayleigh resolution limit. We demonstrate that both the ISM and IES methods are negatively affected by the decrease in object sparsity, and we show the different artifacts that they create when imaging objects of arbitrary shapes. For objects that are largely bright with small dark features, the ISM method is favorable because the intensity fluctuation artifact in IES restorations can make the small dark features invisible. Since the two methods use the same data and differ only in postprocessing, in some cases it may be beneficial for researchers to implement both, particularly when it is suspected that the object has dark features in nonsparse regions.
