# Supplementary material for: Analyzing the super-resolution characteristics of focused-spot illumination approaches
Source: J Biomed Opt. 2020 May 21;25(5):056501. doi: 10.1117/1.JBO.25.5.056501 (PMC7240318; doi:10.1117/1.JBO.25.5.056501)
Supplement: Supplementary file 1 [file JBO_025_056501_SD001.pdf]

Analyzing the super-resolution characteristics of focused-spot illumination approaches: Supplementary Materials

Jiun-Yann Yu[a,b,*], **Venkatalakshmi Narumanchi**[a], **Simeng Chen**[a], **Jian Xing**[a], **Stephen R. Becker**[c], **Carol J. Cogswell**[a]
[a]University of Colorado Boulder, Department of Electrical, Computer and Energy Engineering, Boulder, Colorado, United States, 80309
[b]Current address: 15 Hampton Court, Alhambra, California, United States, 91801
[c]University of Colorado Boulder, Department of Applied Mathematics, Boulder, Colorado, United States, 80309

*Jiun-Yann Yu, jyyu@alumni.caltech.edu

1 Three deconvolution methods for ISM

Here we describe and compare three deconvolution methods that have been applied for ISM establishments: non-negative least-squares (NNLS), Richardson-Lucy (R-L), and Wiener deconvolution (Fig. S1). For NNLS deconvolution, we utilize the 'WPL' method in an ImageJ plugin for iterative deconvolution (Ref. 23 in the main article), and set 'Reflexive' for its boundary, 'Auto' for resizing, 'Double' for precision, 500 for the maximum number of iterations, along with default stopping conditions. For R-L deconvolution, we use MATLAB's `deconvlucy`. With each test object, we run through 10 to 30 R-L iterations and present the best result we find. For Wiener deconvolution, we use the method described by Castello et al. (Ref. 21 in the main article), and we calculate the regularization term η as

$$\eta = \frac{r}{\sqrt{n_{\text{photon}}}},$$

where r is an user-input scalar and n_{photon} the total photon number collected in the pixel-reassigned image I^{ISMimg}. For all of our test objects, the best deconvolution results can be found with r between 20 and 50.

Figure S1(a) shows that the R-L results exhibit the highest visibility, and the Wiener deconvolution results show a slight visibility increase as the SNR increases in the case of a $0.5d_{\text{R}}$ separation, but not as significant as the IES visibility increase shown in Fig. 2(b). While the R-L visibility in Fig. S1(b) is the highest, it also drops most rapidly. Furthermore, in terms of the number of lines, we find that the R-L restorations can sometimes yield incorrect interpretations of the objects, as pointed out by pink arrows in Fig. S1(b). For objects of 4, 5 and 6 lines, the R-L restorations vaguely show 3, 4, and 5 lines instead. In addition, we observe that the R-L restorations incorrectly emphasize the outermost lines in the cases of 3 to 6 lines. This tendency may be the reason for the high visibility of R-L deconvolution observed in Fig. S1(a) and (b). The Wiener deconvolution results, on the other hand, show perceivable ringing artifacts that resemble thin linear structures, as indicated by gray arrows in Fig. S1(b).

In the upper part of Fig. S1(c), both the R-L and Wiener restorations show apparent wormy artifacts at bright regions in the object. In the lower panel, none of the restorations shows clear separations between the hour markings and the clock frame. In the R-L restoration, again we

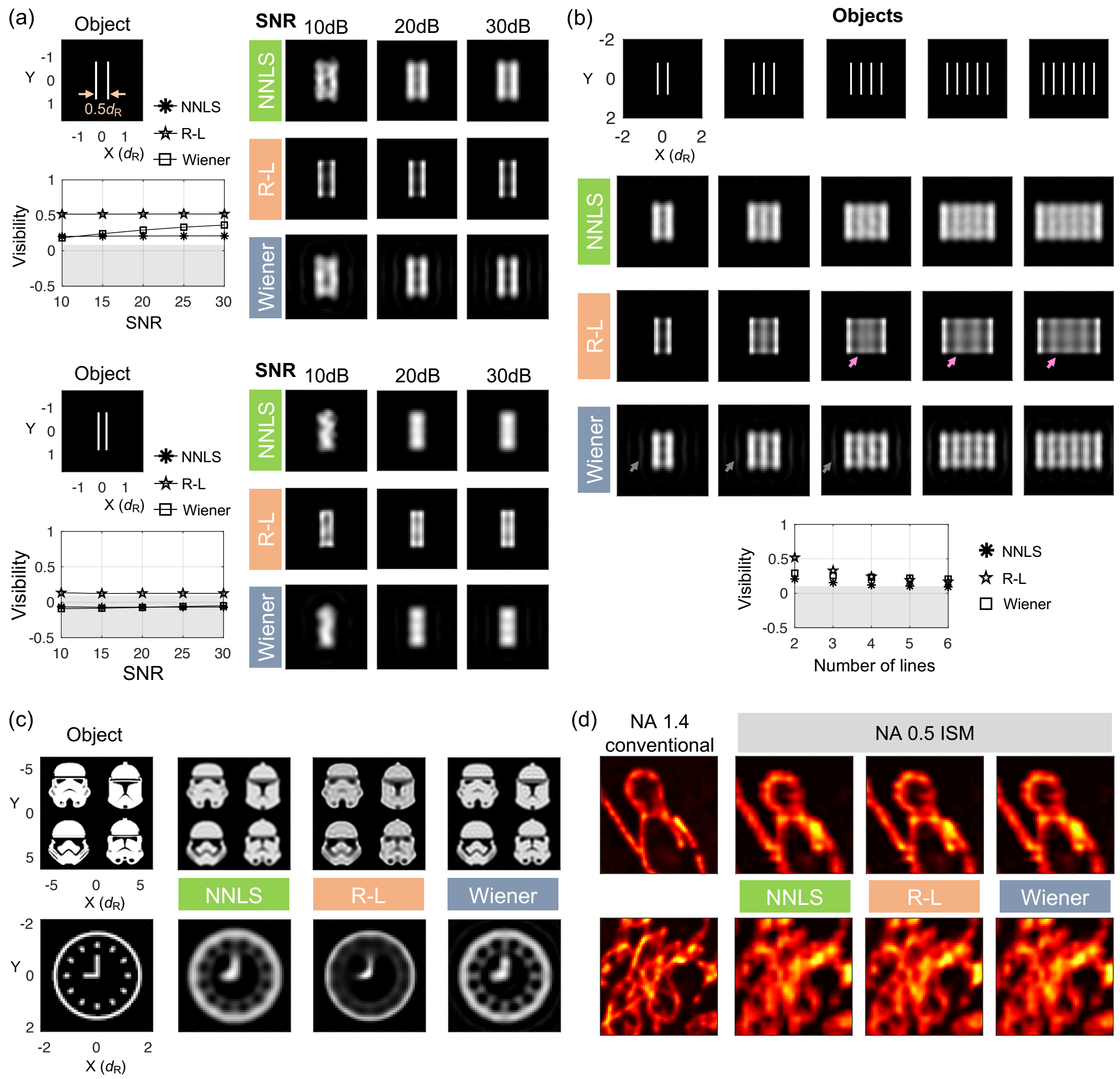

Figure S1 Results of three deconvolution methods for ISM, derived from objects used for Figs. 2-5 in the main article.

observe its emphasis on the outermost object feature, which makes the hour markings almost invisible.

Despite all these differences among the three deconvolution methods we observe using synthetic data, we find that, with careful tuning of the R-L iteration number and the regularization parameter r in Wiener deconvolution, their best results for the biological sample are surprisingly similar, as shown in Fig. S1(d).

We choose to show the results from the NNLS deconvolution in the main figures for two main reasons. Firstly, among all the objects and conditions we test, NNLS deconvolution results are consistently in good quality with minimum artifacts. Secondly and more importantly, the NNLS deconvolution method does not require the user to determine critical parameters, such as the R-L

iteration number and the Wiener regularization parameter, in order to obtain good results.

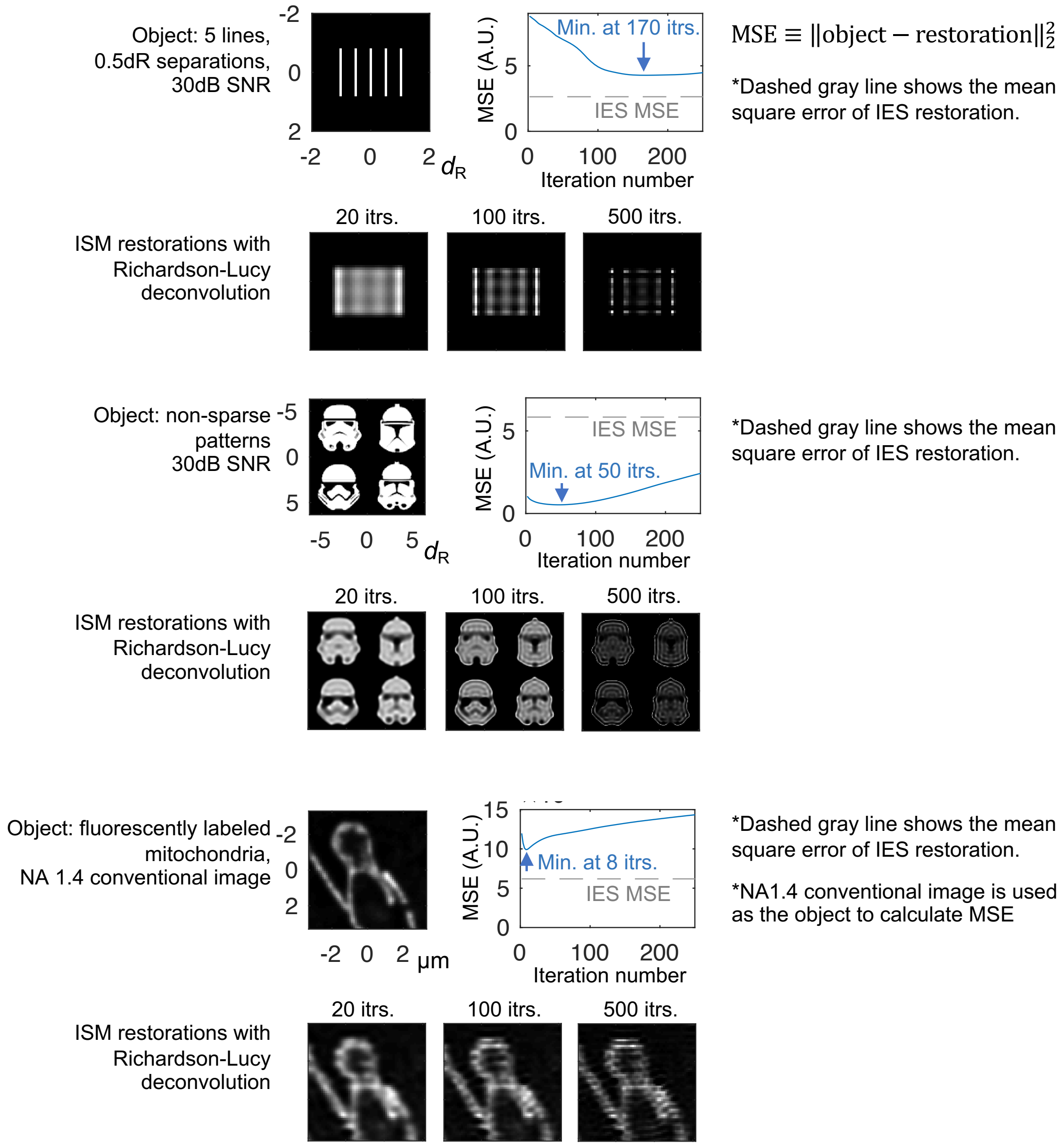

Figure S2 Effect of the R-L iteration number on ISM restorations. The iteration number of the lowest mean square error (MSE) indicates that its restoration matches the object best.

The parameter setting is rather non-trivial in the R-L deconvolution, as shown in Fig. S2. For each test object, we run through 2 to 500 iterations, and calculate the mean square error (MSE) of the restoration at each iteration number. In the MSE plots for the three objects presented in Fig. S2, we find that their optimal iteration numbers, i.e., the iteration numbers that yield the smallest MSE, can differ by nearly two orders of magnitude. In most biomedical applications, because the true structures of the object are usually unknown, determining the optimal iteration number can be technically difficult and oftentimes subjective. In all, we find that the NNLS deconvolution method is simple to utilize, and provides reliable results in most cases.

2 Two-line visibility versus line intensity variation

In Fig. 2 in the main article, we consider only objects of a constant line intensity for simplicity. In reality, however, most fluorescent samples show certain degrees of intensity variation on the object structures, because fluorophore labeling density fluctuates by nature. Since there is no straightforward theoretical approach to estimate the effect of such intensity fluctuation on the ISM and IES methods, we turn to simulations to address this issue. Figure S3 shows the simulation results of the two methods where the line intensity varies at different degrees. In this simulation, we do not observe any significant change in either ISM or IES visibility as we increase the object line intensity variation.

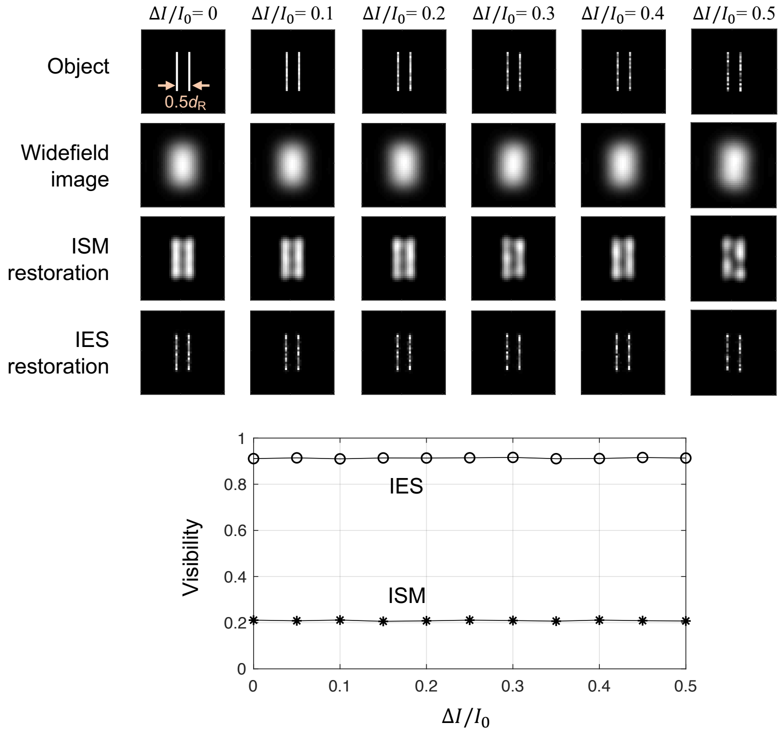

Figure S3 Restoration visibility shows insignificant changes in both the ISM and IES methods. Here ΔI and I_0 are the standard deviation and mean for the intensity of the object lines, respectively. All simulations assume a $0.5d_R$ separation between the two lines and a 20dB SNR, where the SNR is defined by Eq. (9) in the main article.

3 Visibility versus the number of square and circular structures

In the main article, we use linear structures to demonstrate the effect of object sparsity decrease on the restorations of the ISM and IES methods (Fig. 3). To determine whether this effect is universal, we further simulate the restorations for objects of square and circular structures.

The visibility plots in Fig. S4 show a general trend of declining visibility as the objects become less sparse (i.e., having more structures) in both of the structure types, similar to what we observe in Fig. 3 in the main article. We set the the smallest square and circle feature size to be $0.67d_R$, instead of $0.5d_R$ as in the case of Fig. 3, because the ISM restoration does not resolve a $0.5d_R$ circle or square.

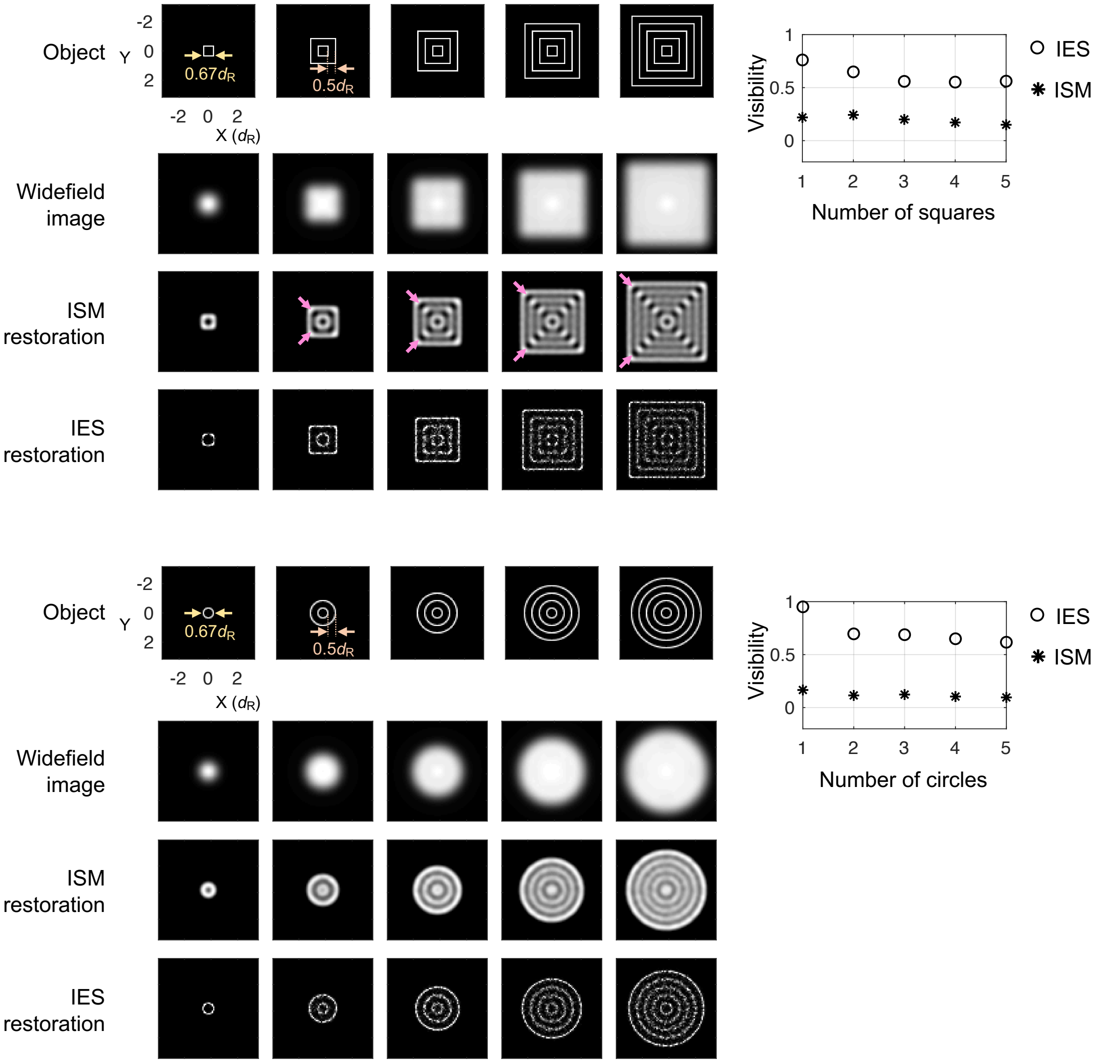

Figure S4 Restoration visibility versus the number of square and circular structures. For both geometrical structures, the smallest one has a feature size of $\sim 0.67d_R$, and each additional structure is set $0.5d_R$ away from and outside of the next largest one. All simulations assume a 20dB SNR, where the SNR is defined by Eq. (9) in the main article.

It is interesting to observe that, for the ISM restorations of circular structures, the smallest circle is only resolved when alone, and becomes unresolved at the presence of outer circles. This again exemplifies that the ISM resolution enhancement is negatively associated with the decrease in object sparsity. Another interesting observation is that the ISM restoration for the single-square object has a visibility slightly lower than its two-square counterpart. We speculate that the cause of this anomaly is the diagonal-band artifacts created in the ISM restorations, as indicated by pink arrows in Fig. S4. When there are multiple squares in the object, the ISM restorations create two diagonal bands with a much higher contrast than the rest of the area. We believe that these high-contrast bands, which do not truthfully resemble the objects, are likely to contribute to the higher visibility for ISM restorations of multiple squares than the case of a single square.

4 Two-line visibility at the presence of a uniform background

According to Eq. (10) in the main article, the object sparsity can drop if there is a sufficiently bright background. Here we examine such an effect on the restoration visibility for two-line objects by adding a uniform background to the objects (Fig. S5).

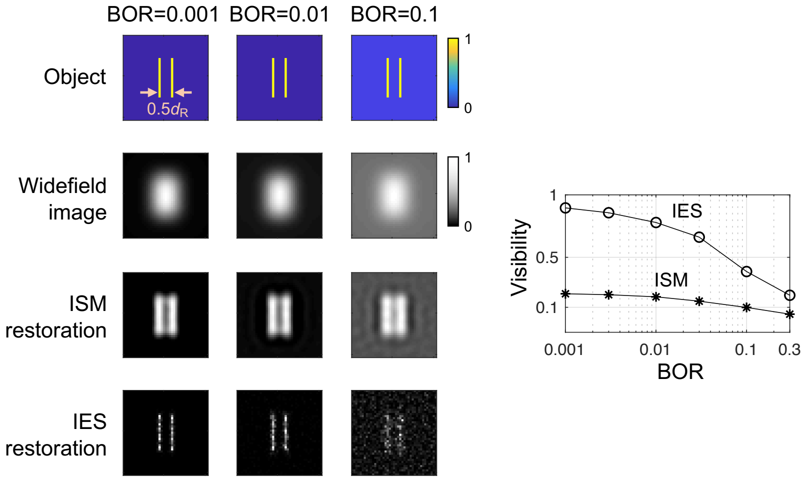

Figure S5 Restoration visibility versus the background-to-object ratio (BOR). All simulations assume a 20dB SNR, where the SNR is defined by Eq. (9) in the main article.

Here we define the background-to-object ratio (BOR) as the intensity ratio of the background to the object lines. The visibility plot in Fig. S5 shows that the both methods exhibit significant visibility drops, especially in the case of IES, as the BOR increases beyond 0.01.

5 Optical system design

We construct the optical system by building a laser scanning module attached to a standard inverted fluorescence microscope (Fig. S6), Zeiss Axiovert 100M. The scanning mirrors (6215H, Cambridge Technology), microscope objective, and two relay lenses are placed in a convemtional 4f arrangement. A 543-nm Helium-Neon laser (LHGR-0100, Research Electro-Optics, Inc.) is used as the excitation light source. The NA0.5 and NA1.4 objectives used for imaging experiments are Zeiss Plan-Neofluar 20X/0.5 and Zeiss Plan-Achromat 63X/1.4, respectively. We use an SCMOS camera (Zyla 5.5, Andor Technology) to acquire images. We develop a LabVIEW application to operate the scanning mirrors and trigger image acquisitions on the camera. We use Micro-Manager as the interface to control the camera.

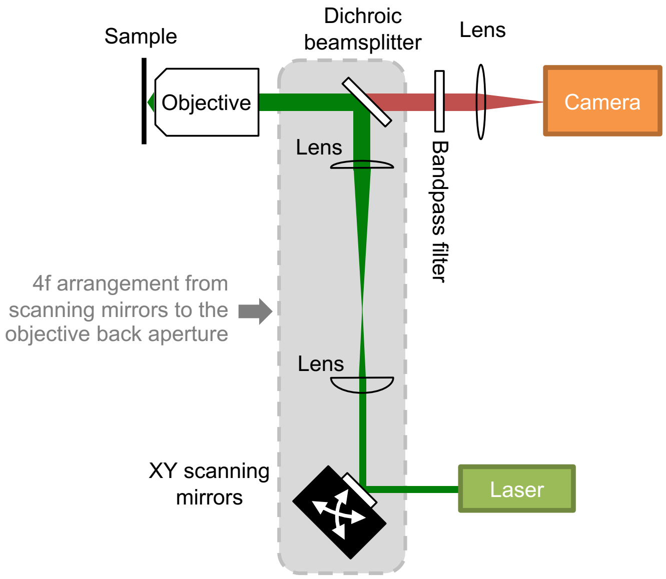

Figure S6 Illustration of the optical system.
